# Discrimination of Clinical and Food-Derived *Candida* Strains Using Biotyping and Molecular Typing Approaches

**DOI:** 10.3390/pathogens14070614

**Published:** 2025-06-20

**Authors:** Katarzyna Rajkowska, Anna Otlewska, Dorota Simińska

**Affiliations:** Institute of Fermentation Technology and Microbiology, Faculty of Biotechnology and Food Sciences, Lodz University of Technology, Wolczanska 171/173, 90-530 Lodz, Poland; d.m.siminska@gmail.com

**Keywords:** *Candida* spp., biotyping, genotyping, ITS region, PCR multiplex, karyotyping, discrimination index, clinical isolates, food-derived strains

## Abstract

Identification and differentiation of *Candida* spp. yeasts, especially clinically relevant isolates, is of high importance with respect to their origin, pathogenic potential, colonization pattern, and resistance to antimycotics. Currently, numerous typing methods with varying or unknown discriminatory power are used. This study evaluated the utility of five methods—biotyping using the API system, ITS1 and ITS4 sequence analysis, ITS region polymorphism, multiplex PCR of ITS1, ITS3, and ITS4 regions, and karyotyping—for typing 42 strains differing in origin (24 clinical and 18 food-borne). The highest discriminatory power was obtained for ITS sequencing and karyotyping, both yielding a discrimination index of 1.000. The discrimination indices for other methods ranged from 0.957 for genotyping based on ITS region polymorphism to 0.997 for multiplex PCR-genotyping. Although biotyping showed relatively high discriminatory potential, its use led to misclassification of 64.3% of isolates compared to ITS sequencing. These findings emphasize the importance of applying a typing method with a discrimination index of 1.000 to ensure accurate interpretation of strain-relatedness and origin. Methods with lower indices may reflect methodological limitations rather than actual genetic relatedness. Determining the discrimination index is therefore essential when selecting appropriate tools for yeast typing, particularly in clinical and epidemiological contexts.

## 1. Introduction

*Candida* yeasts constitute a heterogeneous group of microorganisms capable of colonizing various ecological niches. Due to their clinical importance, research has primarily focused on pathogenic species, with *C. albicans* considered the most important representative [1,2,3]. In addition to *C. albicans*, five non-*albicans Candida* (NAC) species are considered the most clinically relevant, namely *C. glabrata* (current name *Nakaseomyces glabratus*), *C. tropicalis*, *C. parapsilosis*, *C. krusei* (current name *Issatchenkia orientalis*), and the emerging global public health threat *C. auris* [1,2,3,4,5,6]. Invasive *Candida* infections are associated with high morbidity and mortality, as well as increasing antifungal resistance. Invasive candidiasis affects approximately 250,000 to 700,000 individuals worldwide each year, with a mortality rate ranging from 40% to 55% [2,5,6].

It is noteworthy that *Candida* species are predominantly considered opportunistic pathogens, with the majority of infections having an endogenous origin. The pathogenesis of candidiasis is closely related to a number of *Candida* virulence factors, including secretion of hydrolytic enzymes, expression of adhesins and invasins on the cell wall surface, pleomorphism, phenotypic switching, and biofilm formation [2,3]. Furthermore, metabolic plasticity, efficient nutrient acquisition, and a remarkable ability to adapt to environmental stressors contribute significantly to their pathogenic potential.

Different *Candida* species appear to have evolved distinct pathogenic strategies. For instance, *C. albicans* is particularly adept at filamentation and biofilm formation, which contribute to tissue invasion and fungal persistence [3]. In contrast, non-*albicans* species such as *C. glabrata*, *C. parapsilosis*, and *C. auris* exhibit limited filamentation. *C. tropicalis*, on the other hand, demonstrates a higher capacity for biofilm formation and produces more potent candidalysin of cytolytic activity compared to *C. albicans* [3]. Moreover, *C. auris* is more resistant to phagocytosis by neutrophils than *C. albicans*, likely due to the protective properties of its outer cell wall mannan layer [3,7]. Unlike other *Candida* species, *C. glabrata* lacks secreted aspartyl proteases (SAPs) but expresses cell wall-associated aspartic proteases known as yapsins [8]. In addition to these unique virulence traits, antifungal susceptibility remains a key therapeutic concern, with the limited availability of effective treatment options posing a significant challenge in the case of multidrug-resistant species such as *C. auris* and *C. glabrata* [7,8].

Many *Candida* species, including *C. kefyr* (current name *Kluyveromyces marxianus*), *C. boidinii*, *C. lipolytica*, *C. shehatae*, *C. pseudotropicalis*, *C. famata*, *C. guilliermondii* (current name *Meyerozyma guilliermondii*), and *C. inconspicua*, are recognized as part of natural food microbiota, are used in food production, or have been isolated from food products [9,10]. The number of yeasts in these products can reach levels as high as 10^6^–10^8^ CFU/mL or CFU/g [11]. Recent metagenomic data from 2500 food samples demonstrated that *Candida* yeasts occur at low frequency in food environments, with *C. parapsilosis* detected in 2.7% of samples, *C. sake* in 2.5%, and both *Pichia kudriavzevii* (current name *Issatchenkia orientalis*, formerly *Candida krusei*) and *Diutina catenulata* (formerly *C. catenulata*) in 1.3% [12]. In general, yeasts isolated from food that are not part of its native microbiota are considered food spoilage microorganisms.

*Candida* spp. are capable of colonizing the human gastrointestinal tract, contributing to the development of diarrhea and other gastrointestinal symptoms in at-risk individuals. In such cases, fecal samples may contain yeast counts exceeding 10^6^ CFU/g [13]. Talwar et al. [14] identified *C. albicans* as the primary etiologic agent of gastroenteritis, although other species, including *C. tropicalis*, *C. kefyr*, *C. krusei*, *C. parapsilosis*, *C. lusitaniae* (current name *Clavispora lusitaniae*), and *C. guilliermondii*, have also been implicated. In addition, yeasts present in ingested foods may exacerbate Crohn’s disease and induce intestinal inflammatory responses [15]. Nevertheless, gastrointestinal infections caused by food-derived yeast are extremely rare, and only sporadic cases of yeast-induced gastroenteritis have been reported [11].

We have previously shown that food-borne NAC strains may exhibit relevant similarity to clinical *C. albicans*, classifying them within the group of risk of potential pathogens [16]. However, the preliminary and key criterion remains the inability of some food-derived *Candida* spp. strains to grow at human body temperature. To date, no clear evidence has been found to support the transmission of yeasts from food sources to humans. However, the presence of some food-derived *Saccharomyces cerevisiae* isolates in the human gut indicates the potential for yeast transmission through food consumption [12].

Currently, *Candida* spp. infections represent a significant public health problem, highlighting the importance of accurate identification and typing of yeasts in the diagnosis of such infections, as well as in the choice of appropriate antifungal therapy. It also enables the identification of the source of infection and facilitates tracking the development of antifungal drug resistance in epidemiological studies. To identify and distinguish isolates of *Candida* spp. from different sources, numerous molecular typing methods have been proposed, the most recently and commonly used being duplex PCR, restriction fragment length polymorphisms (RFLP), multilocus sequence typing (MLST), randomly amplified polymorphic DNA (RAPD), and microsatellites [17,18,19,20,21]. The utility of these methods has been well documented in previous publications, although literature data concern their application to determine genetic relatedness primarily among clinical *Candida* strains.

The aim of the study was to evaluate the usefulness of various typing methods in the identification and differentiation of *Candida* yeasts isolated from diverse environments, including both clinical and food-related sources. Given their different origins, these strains are likely to vary in virulence and, consequently, in the potential risk they pose to human health [11,16,22]. This study specifically aimed to assess the discriminatory power of selected bio- and genotyping methods that could enhance the accurate differentiation of *Candida* strains from diverse origins. To our best knowledge, most studies to date have focused either on clinical or environmental strains and have not covered such a diverse group of yeasts.

## 2. Materials and Methods

### 2.1. Yeast Strains

The study was conducted on 23 clinical isolates (provided by the Department of Laboratory Diagnostics of the Polish Mother’s Memorial Hospital Research Institute in Lodz, Poland) and 1 reference *Candida albicans* strain ATCC 10231, as well as a group of 18 food-borne strains, including 5 strains from the Culture Collection of Microorganisms LOCK 105 (Table 1). Both the food-derived and clinical *Candida* strains were obtained from diverse sample types and were selected randomly, reflecting the heterogeneity of the source materials used in this study.

### 2.2. Yeast Biotyping

In yeast biotyping, the identification system API 20 C AUX (bioMérieux S.A., Marcy-l’Etoile, France), based on the biochemical assimilation of 19 carbohydrates, was used, according to the manufacturer’s instructions. The API test readings and interpretations were performed independently by two researchers to minimize the potential for bias resulting from subjective assessment. Strain identification was performed on the basis of the obtained numerical profiles, using Apiweb^TM^ software v.1.3.1 (bioMérieux S.A., Marcy-l’Etoile, France).

### 2.3. Sequence Analysis of ITS Regions

Genomic DNA was extracted employing the Genomic Mini AX Yeast (A&A Biotechnology, Gdansk, Poland) in accordance with the manufacturer’s protocol. Spectrophotometric evaluation of DNA was conducted both qualitatively and quantitatively (Implen). DNA at a minimum concentration of 10 ng was used as a template for the PCR reaction. The required DNA purity was determined based on the ratio of absorbance (nm): 260/280 and 260/230, and their values in the ranges of 1.8–2.0 and 2.0–2.2, respectively, were considered acceptable. PCR master mix amplifying the ITS regions contained 12.0 μL of REDTaq Ready Mix polymerase (Sigma-Aldrich, St. Louis, MO, USA), 0.2 μL of ITS1 and ITS4 primers (Genomed Inc., Warsaw, Poland) [23,24] (Table 2), and 10–20 ng of DNA as template (1.0 μL). The PCR was run using the following thermal cycling program: initial denaturation at 94 °C for 2 min, 34 cycles including denaturation at 94 °C for 1 min, annealing at 50 °C for 1 min, elongation at 72 °C for 2 min, and a final extension step at 72 °C for 2 min. The amplicons were separated on 1.0% (*w*/*v*) agarose gel in 0.5× TBE buffer (Sigma-Aldrich, St. Louis, MO, USA) and then purified and sequenced by the Sanger method. The strains were identified by analyzing the sequences (approximately 500 bp in size) using the BLAST+ 2.16.0 (available at https://blast.ncbi.nlm.nih.gov/Blast.cgi (accessed on 7 April 2025)) and then compared with the sequences deposited in the GenBank database (NCBI).

### 2.4. Multiplex PCR Analysis

Multiplex PCR was performed using three universal primers, ITS1, ITS3, and ITS4 (Table 2), targeting the 18S, 5.8S, and 28S rDNA conserved regions [23,24]. To each PCR reaction, 12.0 μL DreamTaq™ Green DNA Polymerase (Thermo Fisher Scientific, Waltham, MA, USA), 0.2 μL of each primer, and 1.0 μL of DNA template were added and made up to a total volume of 25.0 μL with PCR-grade water (Sigma-Aldrich, St. Louis, MO, USA). The multiplex PCR reaction was performed using a thermal cycle as described above, with the primer annealing temperature set at 53 °C. Obtained amplicons were separated on 1.0% (*w*/*v*) agarose gel in 0.5× TBE buffer (Sigma-Aldrich, St. Louis, MO, USA), and the PerfectTM 100 bp DNA Ladder was used as a size standard (EURx, Gdańsk, Poland).

### 2.5. Yeast Karyotyping

Isolation of yeast chromosomal DNA was performed using the CHEF Yeast Genomic DNA Plug Kit (Bio-Rad, Hercules, CA, USA) exactly according to the manufacturer’s instructions. Chromosomes were separated by pulsed-field gel electrophoresis in 0.8% agarose gel (Pulsed-Field Certified Agarose; Bio-Rad, Hercules, CA, USA) by means of CHEF-DR II apparatus (Bio-Rad, Hercules, CA, USA). Electrophoresis was carried out in 0.5× TBE buffer (Sigma-Aldrich, St. Louis, MO, USA) cooled to 10 °C, using the following conditions in two blocks, i.e., block 1: voltage 4.5 V/cm, pulse duration 120 s, separation time 24 h; block 2: voltage 4.5 V/cm, linearly increasing pulse duration from 240 to 360 s, separation time 24 h. After separation, the gels were stained in ethidium bromide solution (50 μg/mL) at room temperature for 15 min [25]. The gels were washed in distilled water at room temperature for 30 min and then photographed. To estimate the molecular weight of chromosomes *Saccharomyces cerevisiae* YNN295 and *Schizosaccharomyces pombe* 972 h DNA size standards (Bio-Rad, Hercules, CA, USA) were used.

### 2.6. Statistical Analysis

#### 2.6.1. Agglomeration Analysis

To determine the similarity of the studied yeast features, the UPGMA cluster analysis (unweighted pair-group method using arithmetic averages) based on the maximum composite likelihood method was used. The similarity analysis of the electrophoretic profiles of ITS regions was performed using GelJ v.2.3 software [26]. Likewise, the karyotype similarity analysis was also conducted employing this software. The dendrogram of the similarity of ITS regions sequences was generated using MEGA 12 program [27]. The procedure for creating dendrogram included 42 nucleotide sequences, and the bootstrap consensus tree was inferred from 500 replicates. The optimal tree with the sum of 5.488 branch length was shown. The pairwise-deletion option was applied to all ambiguous positions for each sequence pair, resulting in a final data set of 693 positions.

#### 2.6.2. Discrimination Index

An index of discrimination (*D*), expressing the ability of a typing method to distinguish between different strains, was calculated according to the Simpson index

(1) $$D=1-\frac{1}{N(N-1)}\sum_{j=1}^{S}n_{j}(n_{j}-1)$$

where *D* is the discrimination index, *N* is the number of tested strains, *s* is the number of types described, and *n_j_* is the number of strains belonging to the *j* type [28]. A *D* value of 1.0 indicates maximal discriminatory power of the method, indicating its ability to uniquely distinguish each individual strain.

## 3. Results

In this study, 42 *Candida* strains were characterized using both biotyping and four genotyping methods. Biotyping was performed based on yeast assimilation capabilities using API tests. The genotyping methods included (1) ITS1 and ITS4 sequence analysis, (2) size polymorphism analysis of the ITS1 and ITS4 regions, (3) multiplex PCR targeting the ITS1, ITS3, and ITS4 regions, and (4) karyotyping. In addition to strain typing, biotyping and ITS sequence-based genotyping were also used to identify the tested strains.

### 3.1. Biotyping and Yeasts Identification by API System

As a result of biotyping, 30 various assimilation profiles were obtained, with 4 profiles shared by more than one strain (Table A1). The first group of yeasts with identical biochemical profiles included five clinical strains: cl/MP/04, cl/MP/07, cl/MP/12, cl/MP/2K, and cl/OZ/g2 (Table 3). The next group consisted of six clinical isolates: cl/MP/02, cl/MP/05, cl/MP/09, cl/MP/4K, cl/MP/3M, and cl/MP/4M. Strains from both of these groups were identified as *C. albicans*. Another assimilation profile was obtained for three food-derived strains: fo/BM/02, fo/MP/02, and LOCK 0009, which were classified as *C. krusei*/*C. inconspicua* (Table 3). The last cluster consisted of two food-borne collection strains: LOCK 0004 and LOCK 0006, identified as *C. lusitaniae*. The other 26 strains were characterized by unique assimilation profiles. Despite differences in the API profiles, 21 clinical isolates and the reference strain ATCC 10231 were identified as *C. albicans*. Only two clinical isolates were classified as non-*albicans Candida* species, namely cl/KL/01 as *C. glabrata* and cl/KL/02 as *C. lusitaniae* (Table 3). Greater species diversity was obtained among food-derived strains. In this group, a total of nine yeast species were identified, i.e., *C. lusitaniae* (four isolates), *C. krusei*/*C. inconspicua* (four), *C. boidinii* (three), *C. famata* (two), *C. parapsilosis* (one), *C. colliculosa* (one), *C. tropicalis* (one), *C. rugosa* (one), and *C. pelliculosa* (one). Notably, in the Apiweb database, strains are reported as *C. krusei*/*C. inconspicua*, indicating taxonomic ambiguity even at high probability levels.

The discrimination index for yeast biotyping based on their assimilation capabilities by means of the API system was equal to 0.966.

### 3.2. Genotyping and Yeast Identification Based on ITS Region Sequences

Partially different results of yeast identification were obtained based on the sequences of the ITS1 and ITS4 regions (Table 3). Reclassification concerned 14 out of 24 clinical isolates and 13 out of 18 food-borne yeasts, representing slightly over 58% and 72% of the strains tested, respectively. This underscores the limited reliability of identification based on API tests alone and highlights the importance of molecular methods in yeast classification.

Moreover, based on API system results, all the tested yeasts were classified within the genus *Candida*, and nearly 89% of clinical isolates were identified as *C. albicans*. In contrast, among the clinical strains, ITS region sequencing revealed two *Candida* species, *C. albicans* (nine strains) and *C. boidinii* (five), whereas the most frequently represented species was *Clavispora lusitaniae* (10 isolates). These findings indicate a possible overidentification of *C. albicans* when using the API system.

Furthermore, isolates classified within the same species differed in their origin. *C. lusitaniae* strains were isolated from feces (five isolates), vagina (two), throat (one), blood (one), and urinary tract (one). The sources of *C. albicans* isolates included feces (six), throat (one), stomach (one), and, in the case of the reference strain ATCC 10231, lungs. Clinical *C. boidinii* strains were obtained from feces (three), stomach (one), and vagina (one).

Among the strains isolated from food, four species belonging to the genus *Candida* were identified (*C. albicans*, *C. lusitaniae*, *C. boidinii*, and *C. tropicalis*), along with four yeast species currently taxonomically reassigned from the *Candida* genus (*Pichia membranifaciens*, *Pichia fermentans*, *Wickerhamomyces anomalus*, and *Meyerozyma guilliermondii*). It is worth emphasizing that strain fo/79/01 isolated from fruit yogurt and fo/BM/01 originating from pickled cucumber were classified as *C. albicans* (Table 3).

Differentiation of yeasts based on the ITS region sequence was characterized by high discriminatory power, as no identical sequences were obtained in the group of the tested strains. The discrimination index for this method reached the highest possible value, amounting to 1.000.

**Figure 1:**
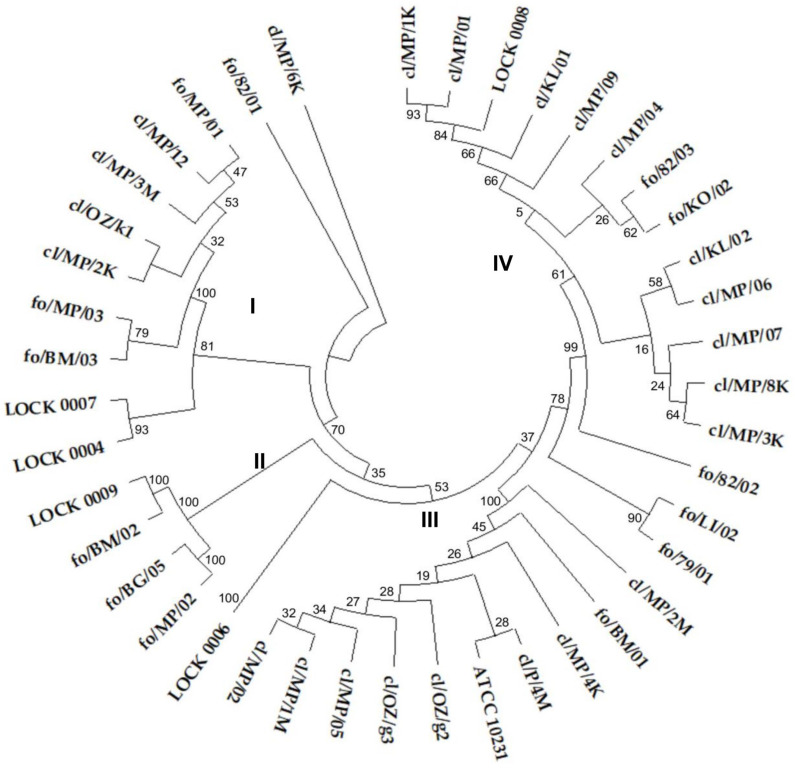
UPGMA cluster analysis tree based on a similarity matrix obtained from ITS1 and ITS4 region sequences of food-borne and clinical yeast isolates. A bootstrap consensus tree was inferred from 500 replicates, and the optimal tree with the sum of 5.488 branch length was shown. The percentage of replicate trees in which the related taxa were clustered together in the bootstrap test is shown next to the branches.

The ITS sequence similarity dendrogram grouped the yeasts into four larger clusters (Figure 1). Cluster I consisted of 7 *C. boidinii* strains, including all three food-derived isolates and four out of five clinical strains classified as this species (cl/MP/12, cl/MP/3M, cl/OZ/k1, and cl/MP/2K), along with single representatives of *M. guilliermondii* (LOCK 0007) and *W. anomalus* (LOCK 0004). Cluster II included two *P. membranifaciens* (fo/MP/02 and fo/BG/05) and two *P. fermentans* isolates (fo/BM/02 and LOCK 0009), all originating from food. Cluster III comprised 10 *C. albicans* strains, representing all clinical isolates of this species included in the study, as well as 1 of the 2 *C. albicans* strains derived from food (fo/BM/01). This cluster is particularly notable for grouping all clinical *C. albicans* strains together, suggesting a high degree of genetic similarity among them. The presence of a single food-derived isolate within this cluster indicates potential overlap between clinical and food-associated *C. albicans* isolates. The second food-borne *C. albicans* strain fo/79/01 showed higher similarity to *C. lusitaniae* fo/LI/02 (Figure 1).

Cluster IV contained 13 strains of *C. lusitaniae*, including all 9 clinical isolates of this species and 3 out of 6 isolated from food (fo/82/03, fo/KO/02, and LOCK 0008). *C. lusitaniae* strain fo/82/01 showed lower similarity to other isolates of this species. On the other hand, the lowest similarity of the ITS region sequence in the tested group of yeasts was exhibited by *C. boidinii* cl/MP/6K (Figure 1). The clustering of yeasts in accordance with their ITS-based species identification reflected a clear congruence between molecular taxonomy and the observed cluster structure. However, it is worth noting the high similarity of clinical and food-borne strains and their frequent common grouping in the same cluster.

Since it seems that more reliable results are obtained using the ITS region sequencing method, the species identification of the tested strains obtained by means of this method was further used.

### 3.3. Yeasts Genotyping Based on ITS Region Polymorphism

**Figure 2:**
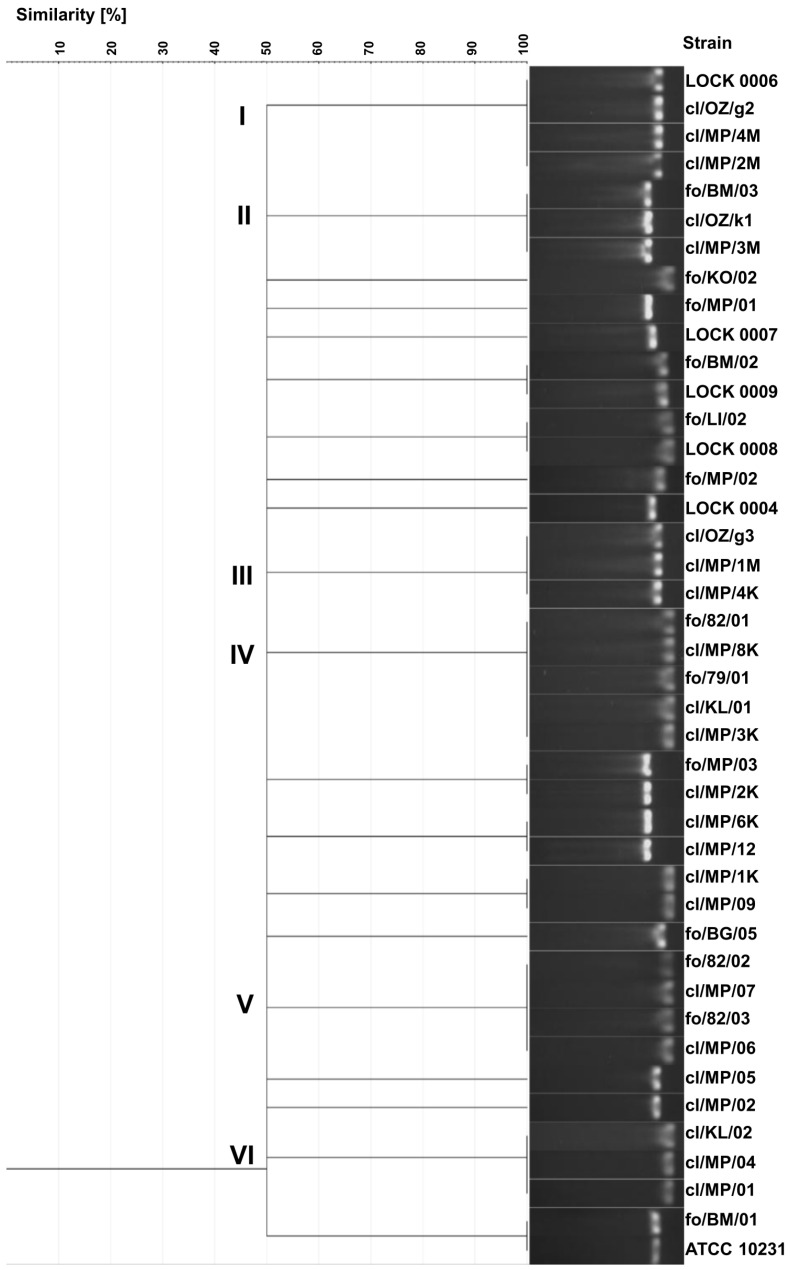
UPGMA dendrogram illustrating the similarity among food-borne and clinical yeast strains based on PCR-amplified ITS region profiles visualized by gel electrophoresis banding patterns.

As a result of electrophoretic separation of PCR products of the ITS region, 20 different electrophoretic profiles were obtained. Identical band sizes were observed in several groups of strains (Figure 2). Identical profiles exhibited three clinical *C. albicans* strains (cl/OZ/g2, cl/MP/4M, and cl/MP/2M) and food-derived *C. tropicalis* LOCK 0006, all showing the band size of 457 bp (cluster I). Cluster II included 3 *C. boidinii* isolates, i.e., two clinical isolates (cl/OZ/k1 and cl/MP/3M) and food-borne strain fo/BM/03, characterized by a band of 526 bp. A different electrophoretic profile with a band size of 463 bp was obtained for three clinical *C. albicans* strains in cluster III (cl/OZ/g3, cl/MP/1M, and cl/MP/4K). Cluster IV included three clinical *C. lusitaniae* isolates (cl/MP/8K, cl/KL/01, and cl/MP/3K), food-derived *C. albicans* fo/79/01, and food-borne *C. lusitaniae* fo/82/01, all of which exhibited a band size of 369 bp. The next cluster consisted of four *C. lusitaniae* strains, including two food-borne strains (fo/82/02 and fo/82/03) and two clinical isolates (cl/MP/07 and cl/MP/06), with an electrophoretic profile characterized by a 384 bp band. Another profile, with a single band of 377 bp, was observed for three clinical *C. lusitaniae* isolates in cluster VI (cl/KL/02, cl/MP/04, and cl/MP/01).

In addition, identical electrophoretic profiles were obtained for the following pairs of strains: *P. fermentans* fo/BM/02 and LOCK 0009, *C. lusitaniae* fo/LI/02 and LOCK 0008, *C. boidinii* fo/MP/03 and cl/MP/2K, *C. boidinii* cl/MP/6K and cl/MP/12, *C. lusitaniae* cl/MP/1K and cl/MP/09, and *C. albicans* fo/BM/01 and ATCC 10231. Only eight strains (*C. lusitaniae* fo/KO/02, *C. boidinii* fo/MP/01, *W. anomalus* LOCK 0007, *P. membranifaciens* fo/MP/02 and fo/BG/05, *M. guilliermondii* LOCK 0004, *C. albicans* cl/MP/05, and cl/MP/02) exhibited unique electrophoretic profiles of ITS region PCR products (Figure 2).

A total of 20 distinct electrophoretic profiles were obtained using this method, with yeasts of the same species generally clustering together. However, four electrophoretic profiles were indistinguishable among isolates from different species, indicating a limitation in the method’s taxonomic utility. Furthermore, clinical and food-derived strains were frequently grouped within the same cluster. The discrimination index of the yeast genotyping method based on ITS region polymorphism, relying on electrophoretic separation of the PCR product, was the lowest among all methods used, amounting to 0.957. This reduced discriminatory power may be attributable to limited resolution in band size differentiation and low strain heterogeneity. Electrophoretic profiles were scored with software support (GelJ), which was used for both band calling and cluster analysis.

### 3.4. Genotyping Based on Multiplex PCR

As a result of the multiplex PCR analysis, 39 different electrophoretic profiles were obtained, demonstrating greater diversity than that observed in the ITS region polymorphisms analysis. The higher discriminatory power of this method resulted from the increased complexity of band patterns generated in multiplex PCR, which captures polymorphisms across multiple loci, compared to the single band produced by ITS region-based genotyping.

**Figure 3:**
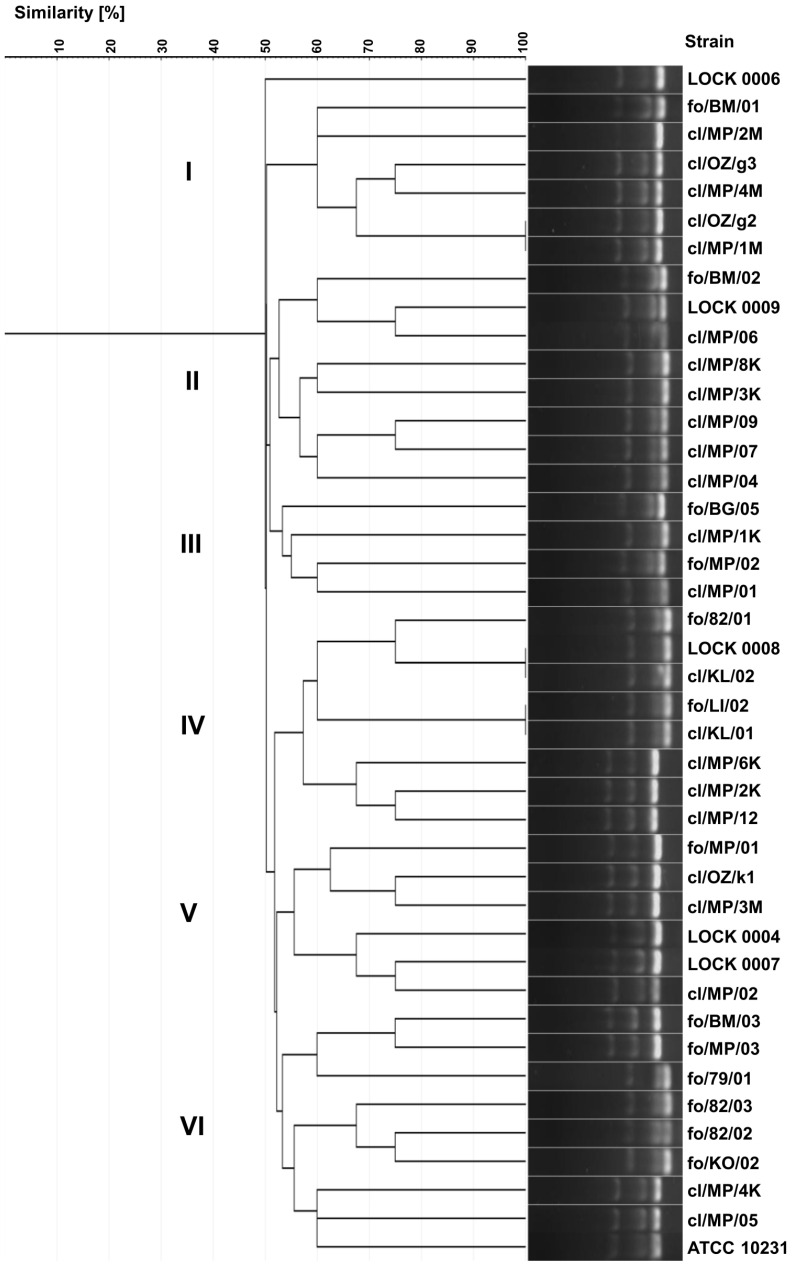
UPGMA dendrogram illustrating the similarity of food-borne and clinical yeast profiles generated by multiplex PCR targeting the 18S, 5.8S, and 28S rDNA regions and visualized as electrophoretic banding patterns.

Identical electrophoretic profiles were noted for only three pairs of strains, namely *C. albicans* cl/OZ/g2 and cl/MP/1M, *C. lusitaniae* LOCK 0008 and cl/KL/02, and *C. lusitaniae* fo/LI/02 and cl/KL/01 (Figure 3). Yeast strains were grouped into six internally diversified clusters, within which the similarity of electrophoretic profiles ranged from 50% to 100%. Cluster I included six strains belonging exclusively to one species, *C. albicans*, including five clinical isolates (cl/MP/1M, cl/OZ/g2/, cl/MP/4M, cl/OZ/g3, and cl/MP/2M) and food-borne fo/BM/01. Cluster II consisted of six clinical *C. lusitaniae* isolates (cl/MP/04, cl/MP/07, cl/MP/09, cl/MP/3K, cl/MP/8K, and cl/MP/06) and two food-derived *P. fermentans* strains (LOCK 0009 and fo/BM/02). Cluster III comprised both strains of *P. membranifaciens* originating from food (fo/BG/05 and fo/MP/02), as well as two clinical isolates of *C. lusitaniae* (cl/MP/1K and cl/MP/01). Cluster IV grouped together three clinical *C. boidinii* isolates (cl/MP/12, cl/MP/2K, and cl/MP/6K) and five *C. lusitaniae*, i.e., two clinical (cl/KL/01 and cl/KL/02) and three originating from food (fo/LI/02, LOCK 0008, and fo/82/01). Cluster V was the most diverse and contained three *C. boidinii* strains of various origins (cl/MP/3M, cl/OZ/k1, and fo/MP/01), clinical *C. albicans* cl/MP/02, as well as food-derived *W. anomalus* LOCK 0007 and *M. guilliermondii* LOCK 0004. Cluster VI was dominated by food-derived yeasts, namely *C. lusitaniae* fo/KO/02, fo/82/02, and fo/82/03, *C. albicans* fo/79/01, as well as *C. boidinii* fo/BM/03 and fo/MP/03. This group also included two clinical isolates of *C. albicans* (cl/MP/05 and cl/MP/4K) and the collection strain ATCC 10231 (Figure 3).

In multiplex PCR-based genotyping, as previously observed, both clinical and food-borne strains were grouped into the same clusters. Strains representing different species were also grouped together, which may reflect strain convergence. Notably, identical electrophoretic profiles were obtained only for yeasts classified within the same species. The discrimination index of the yeast genotyping method based on electrophoretic separation of multiplex PCR products was 0.997, and it was higher than the indices of both biotyping and ITS region-based genotyping.

### 3.5. Yeasts Karyotyping

**Figure 4:**
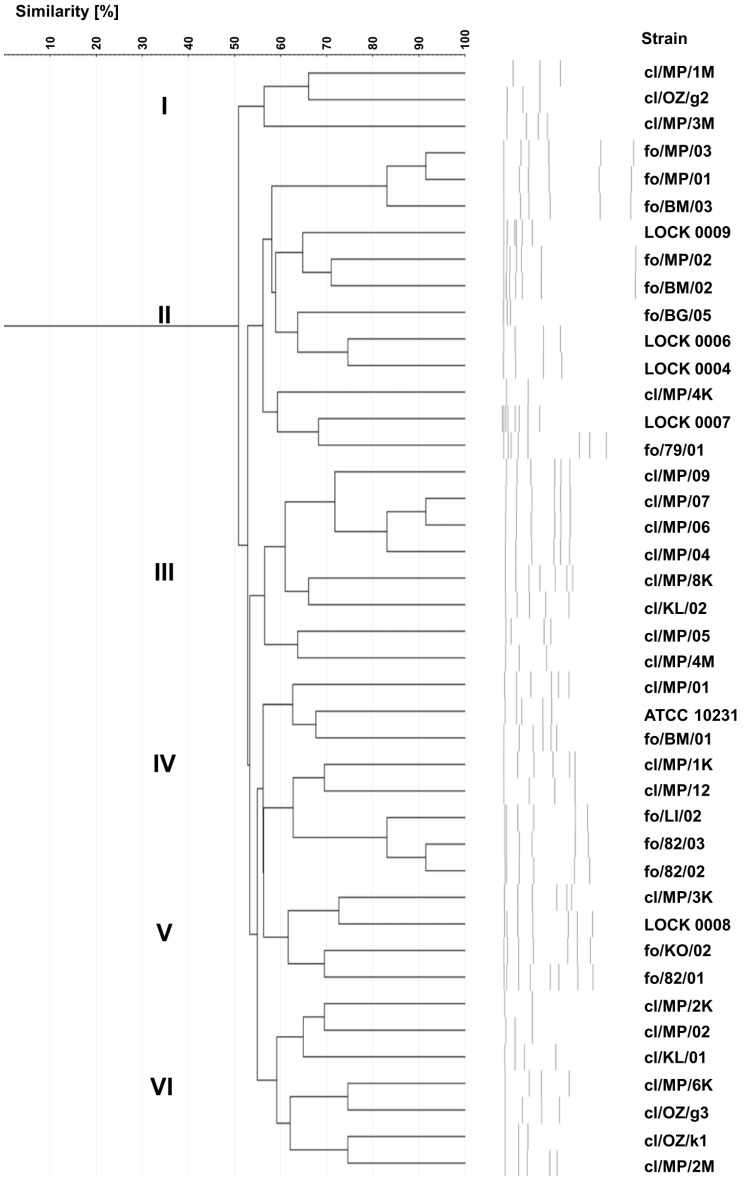
UPGMA dendrogram showing the similarity of electrophoretic karyotypes of food-borne and clinical yeast strains.

Karyotyping revealed 42 different electrophoretic profiles of yeast chromosomal DNA (Figure 4), indicating that each of the strains tested exhibited a unique karyotype. The highest profile similarity observed between any two strains was ~90% and was observed in three pairs of yeasts, i.e., two food-derived *C. boidinii* strains (fo/MP/03 and fo/MP/01), two clinical *C. lusitaniae* isolates (cl/MP/07 and cl/MP/06), and two *C. lusitaniae* strains originating from food (fo/82/03 and fo/82/02).

On the dendrogram, 75% of clinical strains were grouped into three clusters—cluster I, III, and VI—composed only of clinical isolates. However, in terms of species composition, all three clusters were internally diverse. Cluster I comprised two *C. albicans* isolates (cl/MP/1M and cl/OZ/g2) and *C. boidinii* cl/MP/3M. Cluster III included 6 out of 10 *C. lusitaniae* strains (cl/MP/09, cl/MP/07, cl/MP/06, cl/MP/04, cl/MP/8K, and cl/KL/02), along with two *C. albicans* isolates (cl/MP/05 and cl/MP/4M). Cluster VI encompassed three *C. boidinii* strains (cl/MP/2K, cl/MP/6K, and cl/OZ/k1), three *C. albicans* isolates (cl/MP/02, cl/OZ/g3, and cl/MP/2M), and *C. lusitaniae* cl/KL/01 (Figure 4). In cluster II, apart from *C. albicans* cl/MP/4K, food-derived strains were grouped, namely *C. albicans* fo/79/01, as well as all food-borne strains identified as *C. boidinii* (fo/BM/03, fo/MP/01, and fo/MP/03), *P. fermentans* (fo/BM/02 and LOCK 0009), *P. membranifaciens* (fo/BG/05 and fo/MP/02), *C. tropicalis* (LOCK 0006), *M. guilliermondii* (LOCK 0004), and *W. anomalus* (LOCK 0007). Cluster IV was the most diverse, containing both clinical and food-borne strains of *C. lusitaniae* (cl/MP/01, cl/MP/1K, fo/LI/02, fo/82/03, and fo/82/02), *C. albicans* (fo/BM/01 and ATCC 10231), and *C. boidinii* cl/MP/12. In contrast, cluster V included only *C. lusitaniae* strains, but differing in origin, i.e., three food-derived yeasts (LOCK 0008, fo/KO/02, and fo/82/01) and one clinical isolate cl/MP/3K.

Karyotyping, similarly to genotyping based on ITS sequences, proved to be a highly effective method for typing yeast, with the discrimination index of 1.000. Among the applied methods, karyotyping appeared to allow the most structured and coherent grouping of yeasts according to their origin.

## 4. Discussion

The evaluation of the effectiveness of yeast typing methods is typically based on several parameters, i.e., typeability, reproducibility, and discriminatory power [28]. Among these characteristics, in this study we focused on the discriminatory power of typing methods, which is defined as the ability to distinguish between unrelated strains [28]. From both clinical and epidemiological points of view, but also from a scientific perspective, differentiation of *Candida* spp. strains in terms of their origin is crucial, particularly in relation to sources of infection, yeast’s pathogenic potential, colonization patterns, resistance to antimycotics, as well as strain microevolution within species [4,6,18,19,29].

High discriminatory power does not always imply clinical relevance or reproducibility. The usefulness of numerous methods in this regard has been well described in previous publications [17,18,19,20,21,29]. Nevertheless, comparing the discriminatory power of different typing methods is difficult since the discrimination index was rarely reported. Available data indicate that the discrimination index for the microsatellites method may be 0.85–0.91 [18]; for DNA typing, 0.868 [28]; for RAPD fingerprinting, 0.984; and for karyotyping, only 0.630 [30]. It is assumed that for typing results to be interpreted with confidence, the discrimination index should be greater than 0.90 [28].

In this study, discrimination indices were determined for five selected typing methods, namely biotyping based on yeast assimilation profiles using the popular and commonly used API system, genotyping based on ITS region polymorphism and ITS sequencing, multiplex PCR of ITS regions, and karyotyping. The highest discriminatory capacity (*D* = 1.000), enabling differentiation of all *Candida* spp. strains tested, was achieved through karyotyping and ITS sequence analysis. The multiplex PCR method demonstrated very high discriminatory ability (*D* = 0.997), while biotyping showed lower discriminatory power (*D* = 0.966). ITS region-based genotyping was the least discriminatory among the methods evaluated, with a discrimination index of 0.957. However, despite the differences in the index values, all tested typing methods fulfilled the criterion for results to be interpreted with confidence [28]. On the other hand, a high index does not guarantee correct species identification, as demonstrated by the API 20C AUX system, which exhibited a misclassification rate of 64.3%. It should also be noted that the discrimination index value strongly depends on the number and diversity of strains analyzed.

Among the methods used in this study, karyotyping is also applicable for yeast identification. However, this method is challenging for species identification due to yeast chromosomal polymorphism, genomic instability, intraspecies variation, aneuploidy, and the occurrence of co-migrating bands [31]. Its primary utility lies in strain-level differentiation [31], which was confirmed by our results, as each isolate exhibited a distinct karyotype even among conspecific strains. Therefore, the utility of karyotyping is primarily in distinguishing strains rather than in species identification.

According to ITS sequence-based identification, a significantly higher species diversity was observed among both food-derived and clinical yeasts compared to biotyping results. Among the clinical strains, in addition to *C. albicans* and *C. lusitaniae*, isolates belonging to *C. boidinii* were also identified, with *C. lusitaniae* being the predominant species. This finding is partly consistent with previous reports on NAC species associated with human infections [6,17,32]. However, *C. boidinii* is rarely mentioned in this context, although clinical strains of this species have been reported [33]. Similarly, among food-borne yeasts, the identification of two strains as *C. albicans* is surprising, as this species has not been previously associated with food sources. Other strains identified among food-derived yeasts, i.e., *W. anomalus* and *C. lusitaniae*, are also considered opportunistic pathogens that may cause infections, especially in people at risk [17,32,34].

These findings are consistent with previous studies suggesting the inability to distinguish between clinical and environmental strains. In line with this, no genetic distinction was found between 20 clinical *C. krusei* isolates and 12 environmental *P. kudriavzevii* isolates, indicating that these yeasts belong to the same species [35]. High genetic congruence was also observed for yeasts originating from different environments in various regions of Mumbai (soil adjacent to urinals, sewage water, beach water, and hospital soil), and among the identified species, *C. albicans*, *C. tropicalis*, and *C. krusei* were found in descending order of abundance [19]. Moreover, environmental and food-borne strains may exhibit similar levels of drug resistance to clinically relevant isolates [19,35,36]. Douglass et al. [35] further hypothesize that food-borne or environmental exposure may contribute to colonization or infection in vulnerable individuals.

These findings are consistent with the One Health concept, which assumes the potential transmission of microorganisms between animals, humans, and ecosystems, with a particular focus on emerging and endemic zoonoses [37]. Antimicrobial resistance also remains a critical concern, as resistance can arise in humans, animals, or the environment and may spread between these reservoirs. *Candida* strains that were thermotolerant to human body temperature, pathogenic, and resistant to at least one antifungal drug were detected on plastic pollutants in aquatic ecosystems [38]. These plastic pollutants may serve as important vehicles for the dissemination of human pathogens and the potential exchange of antimicrobial resistance genes across various ecosystems, including terrestrial environments [39,40]. The significance of the natural environment in the epidemiology of infectious diseases—as a source of pathogens—was documented for *C. auris*, a recently emerged human pathogenic yeast. Environmental reservoirs of antibiotic-resistant *C. auris* include terrestrial, freshwater, and marine ecosystems, with colonized humans or animals or contaminated clinical waste, likely serving as sources of environmental contamination [41]. Other pathogenic *Candida* species—including *C. albicans*, *C. glabrata*, *C. dubliniensis*, *C. krusei*, and *C. parapsilosis*—were detected at various stages of wastewater treatment [42], further reinforcing the One Health paradigm.

The phenomenon of microorganism transmission between different environments may partly explain the high similarity of strains of diverse origins observed in this study, as well as the clustering of food-borne and clinical isolates within the same groups.

## 5. Conclusions

This study contributes to a better understanding of the discriminatory capacity and limitations of various typing methods used for yeast identification and strain differentiation. The highest discriminatory power (*D* = 1.000) for distinguishing *Candida* spp. strains from diverse sources was observed with genotyping based on ITS region sequencing and karyotyping. Identification using ITS sequences proved significantly more reliable than that based on assimilation profiles using the API system. Our findings highlight the need for a cautious interpretation of the discrimination index, particularly when *D* < 1.000 may reflect methodological limitations rather than true strain identity. The fact that all strains were differentiated by two out of five tested methods suggests distinct strain origins and highlights the importance of applying at least one typing method with a *D* = 1.000 in such studies. This approach minimizes the risk of misinterpreting strain-relatedness and origin. In this context, our findings may provide practical recommendations for researchers and clinicians when selecting appropriate tools for taxonomic and epidemiological investigations.

On the other hand, the results of this study should be regarded as preliminary, emphasizing the relevance of the discriminatory index. Very high D values may sometimes lead to an overestimation of diversity due to a method’s sensitivity to minor, potentially irrelevant variations. Moreover, this study focused primarily on discriminatory power, without including detailed phylogenetic or clinical outcome analyses. Future research should involve broader comparative approaches, including, for example, whole-genome sequencing of yeast strains. Integrating complementary techniques—such as combining molecular and phenotypic methods—may further improve the accuracy and reliability of yeast identification and strain typing.

Our primary objective was to draw attention to the potential for misinterpretation that may arise from methodological limitations. Moreover, these findings support the growing recognition of genetic-relatedness among *Candida* strains originating from varied environments, which has implications for infection control, outbreak investigation, and microbial ecology within the One Health framework.

## Figures and Tables

**Table 1:** Yeast strains used in the study.

Strain	Source of Isolation
clinical strains	
cl/MP/01, cl/MP/02, cl/MP/12, cl/MP/1K, cl/MP/3K, cl/MP/4K, cl/MP/8K, cl/MP/1M, cl/MP/2M, cl/MP/3M, cl/MP/4M, cl/OZ/g2, cl/OZ/k1, cl/KL/02	faces
cl/MP/04, cl/OZ/g3	throat
cl/MP/05, cl/MP/2K	stomach
cl/MP/06, cl/MP/07, cl/MP/6K	vagina
cl/MP/09	blood
cl/KL/01	urinary tract
*C. albicans* ATCC 10231	lungs
food-borne strains	
fo/79/01, fo/82/01, fo/82/02, fo/82/03, fo/KO/02	fruit yogurt
fo/LI/02	herring salad
fo/MP/01, fo/MP/02, fo/MP/03, fo/BM/01, fo/BM/02, fo/BM/03	pickled cucumbers
fo/BG/05	sauerkraut
LOCK 0004, LOCK 0006, LOCK 0007	feed yeast
LOCK 0008, LOCK 0009	baker’s yeast

**Table 2:** Sequences of primers used in this study.

Primer	Primer Sequence	Reference
ITS1	5′–TCCGTAGGTGAACCTGCGG–3′	[23]
ITS3	5′–GCATCGATGAAGAACGCAGC–3′	[24]
ITS4	5′–TCCTCCGCTTATTGATATGC–3′	[23,24]

**Table 3:** Identification of clinical and food-borne yeasts. If different, current species names according to www.indexfungorum.org (accessed on 4 June 2025) are given in parentheses. Strains that were differently classified into species based on assimilation profiles (API 20 C AUX) and ITS region sequences are in bold.

Strain	Source of Isolation	Identification Based on Assimilation Profiles	Identification Based on ITS Sequence	Accession Number ^1^
clinical strains				
cl/MP/02	Faces	*Candida albicans*	*Candida albicans*	PV670445
cl/MP/4K	Faces	*Candida albicans*	*Candida albicans*	PV670447
cl/MP/1M	Faces	*Candida albicans*	*Candida albicans*	PV670444
cl/MP/2M	Faces	*Candida albicans*	*Candida albicans*	PV670446
cl/MP/4M	Faces	*Candida albicans*	*Candida albicans*	PV670448
cl/OZ/g2	Faces	*Candida albicans*	*Candida albicans*	PV670450
cl/OZ/g3	Throat	*Candida albicans*	*Candida albicans*	PV670451
cl/MP/05	stomach	*Candida albicans*	*Candida albicans*	PV670449
ATCC 10231	Lungs	*Candida albicans*	*Candida albicans*	PV785352
**cl/MP/01**	Faces	*Candida albicans*	*Clavispora lusitaniae*	PV670459
**cl/MP/1K**	Faces	*Candida albicans*	*Clavispora lusitaniae*	PV670460
**cl/MP/3K**	Faces	*Candida albicans*	*Clavispora lusitaniae*	PV670461
**cl/MP/8K**	Faces	*Candida albicans*	*Clavispora lusitaniae*	PV670465
**cl/MP/04**	Throat	*Candida albicans*	*Clavispora lusitaniae*	PV670462
**cl/MP/06**	Vagina	*Candida albicans*	*Clavispora lusitaniae*	PV670463
**cl/MP/07**	Vagina	*Candida albicans*	*Clavispora lusitaniae*	PV670464
**cl/MP/09**	Blood	*Candida albicans*	*Clavispora lusitaniae*	PV670466
cl/KL/02	Faces	*Candida lusitaniae* (*Clavispora lusitaniae*)	*Clavispora lusitaniae*	PV670458
**cl/KL/01**	urinary tract	*Candida glabrata* (*Nakaseomyces glabratus*)	*Clavispora lusitaniae*	PV670457
**cl/MP/12**	Faces	*Candida albicans*	*Candida boidinii*	PV670455
**cl/MP/3M**	Faces	*Candida albicans*	*Candida boidinii*	PV670453
**cl/OZ/k1**	Faces	*Candida albicans*	*Candida boidinii*	PV670456
**cl/MP/2K**	stomach	*Candida albicans*	*Candida boidinii*	PV670452
**cl/MP/6K**	Vagina	*Candida albicans*	*Candida boidinii*	PV670454
food-borne strains				
fo/82/01	fruit yogurt	*Candida lusitaniae* (*Clavispora lusitaniae*)	*Clavispora lusitaniae*	PV686768
**fo/82/02**	fruit yogurt	*Candida famata* (*Debaryomyces hansenii*)	*Clavispora lusitaniae*	PV686769
**fo/82/03**	fruit yogurt	*Candida parapsilosis*	*Clavispora lusitaniae*	PV686770
**fo/KO/02**	fruit yogurt	*Candida colliculosa*	*Clavispora lusitaniae*	PV686771
**fo/LI/02**	herring salad	*Candida famata* (*Debaryomyces hansenii*)	*Clavispora lusitaniae*	PV686772
**LOCK 0008**	baker’s yeast	*Candida krusei* (*Issatchenkia orientalis*)/*C. inconspicua* (*Pichia inconspicua*)	*Clavispora lusitaniae*	PV686773
**fo/79/01**	fruit yogurt	*Candida lusitaniae* (*Clavispora lusitaniae*)	*Candida albicans*	PV686762
**fo/BM/01**	pickled cucumbers	*Candida tropicalis*	*Candida albicans*	PV686763
fo/MP/01	pickled cucumbers	*Candida boidinii*	*Candida boidinii*	PV686765
fo/MP/03	pickled cucumbers	*Candida boidinii*	*Candida boidinii*	PV686766
fo/BM/03	pickled cucumbers	*Candida boidinii*	*Candida boidinii*	PV686764
**LOCK 0006**	feed yeast	*Candida lusitaniae* (*Clavispora lusitaniae*)	*Candida tropicalis*	PV686767
**fo/MP/02**	pickled cucumbers	*Candida krusei* (*Issatchenkia orientalis*)/*C. inconspicua* (*Pichia inconspicua*)	*Pichia membranifaciens*	PV686778
**fo/BG/05**	sauerkraut	*Candida rugosa* (*Diutina rugosa*)	*Pichia membranifaciens*	PV686777
**fo/BM/02**	pickled cucumbers	*Candida krusei* (*Issatchenkia orientalis*)/*C. inconspicua* (*Pichia inconspicua*)	*Pichia fermentans*	PV686775
**LOCK 0009**	baker’s yeast	*Candida krusei* (*Issatchenkia orientalis*)/*C. inconspicua* (*Pichia inconspicua*)	*Pichia fermentans*	PV686776
**LOCK 0004**	feed yeast	*Candida lusitaniae* (*Clavispora lusitaniae*)	*Meyerozyma* *guilliermondii*	PV686774
LOCK 0007	feed yeast	*Candida pelliculosa* (*Wickerhamomyces* *anomalus*)	*Wickerhamomyces* *anomalus*	PV686779

^1^ Accession numbers in the GeneBank NCBI database based on nucleotide sequences of the ITS regions.

## Data Availability

All nucleotide sequences of the ITS regions analyzed during the current study are available in the GeneBank NCBI database under the accession numbers PV670444 to PV670466, PV785352, and PV686762 to PV686779.

## Appendix A

**Table A1:** API profiles of clinical and food-borne yeast strains.

Substrate ^1^	1	2	3	4	5	6	7	8	9	10	11	12	13	14	15	16	17	18	19
cl/MP/02	+	−	+	−	+	+	+	+	−	+	+	+	−	−	+	+	+	−	−
cl/MP/4K	+	−	+	−	+	+	+	+	−	+	+	+	−	−	+	+	+	−	−
cl/MP/1M	+	+	+	−	+	+	+	+	+	+	+	+	−	−	+	+	+	−	−
cl/MP/2M	+	−	+	−	−	+	+	+	−	+	+	+	−	−	+	+	+	−	−
cl/MP/4M	+	−	+	−	+	+	+	+	−	+	+	+	−	−	+	+	+	−	−
cl/OZ/g2	+	−	+	−	+	−	+	+	−	+	+	+	−	−	+	+	+	−	−
cl/OZ/g3	+	−	+	−	+	+	+	+	−	+	+	+	−	−	+	+	−	−	−
cl/MP/05	+	−	+	−	+	+	+	+	−	+	+	+	−	−	+	+	+	−	−
ATCC 10231	+	−	+	−	+	+	+	+	+	+	+	+	−	−	+	+	+	−	−
cl/MP/01	+	+	+	+	+	+	+	+	−	+	+	+	−	−	+	+	+	−	−
cl/MP/1K	+	+	+	−	+	−	+	+	−	+	+	+	−	−	+	+	+	−	−
cl/MP/3K	+	−	+	−	−	+	+	+	+	+	+	+	−	−	+	+	+	−	−
cl/MP/8K	+	−	+	−	−	+	+	+	+	+	−	+	−	−	+	+	+	−	−
cl/MP/04	+	−	+	−	+	−	+	+	−	+	+	+	−	−	+	+	+	−	−
cl/MP/06	+	−	+	−	−	+	+	+	−	+	−	+	−	−	+	+	+	−	−
cl/MP/07	+	−	+	−	+	−	+	+	−	+	+	+	−	−	+	+	+	−	−
cl/MP/09	+	−	+	−	+	+	+	+	−	+	+	+	−	−	+	+	+	−	−
cl/KL/02	+	+	+	−	+	+	+	+	−	+	−	−	+	−	+	+	+	+	−
cl/KL/01	+	−	+	−	−	−	−	−	−	−	−	−	−	−	−	−	+	−	−
cl/MP/12	+	−	+	−	+	−	+	+	−	+	+	+	−	−	+	+	+	−	−
cl/MP/3M	+	−	+	−	+	+	+	+	−	+	+	+	−	−	+	+	+	−	−
cl/OZ/k1	+	+	+	−	+	−	+	+	−	+	+	+	−	−	+	+	−	−	−
cl/MP/2K	+	−	+	−	+	−	+	+	−	+	+	+	−	−	+	+	+	−	−
cl/MP/6K	+	+	+	−	+	+	+	+	−	+	+	+	−	−	+	+	+	−	−
fo/82/01	+	+	+	−	+	−	−	−	−	+	+	+	+	−	+	+	+	+	−
fo/82/02	+	+	+	−	−	+	+	+	−	+	+	+	+	+	+	+	+	+	−
fo/82/03	+	+	+	+	−	+	−	+	−	+	−	+	−	−	+	+	+	+	−
fo/KO/02	+	+	+	−	−	−	+	−	−	+	−	−	−	−	−	+	+	−	+
fo/LI/02	+	+	+	−	−	+	+	+	−	+	+	+	+	+	+	+	−	+	+
LOCK 0008	+	−	−	−	+	−	−	−	−	−	−	+	−	−	−	−	−	−	−
fo/79/01	+	+	+	−	−	−	−	+	−	+	−	+	+	−	+	+	−	+	−
fo/BM/01	+	−	+	−	+	+	−	+	−	+	−	+	−	−	+	−	+	−	−
fo/MP/01	+	+	−	−	+	+	+	−	−	+	−	+	−	−	−	+	−	−	−
fo/MP/03	+	+	−	−	+	+	+	−	−	+	−	+	−	−	−	−	−	−	−
fo/BM/03	+	+	−	−	+	+	+	−	−	+	−	+	+	−	−	−	−	−	+
LOCK 0006	+	+	+	−	+	+	+	+	+	+	+	+	+	−	+	+	+	+	−
fo/MP/02	+	+	−	−	+	−	−	−	−	−	−	+	−	−	−	−	−	−	−
fo/BG/05	+	−	−	−	+	−	−	+	−	+	−	−	−	−	−	−	−	−	−
fo/BM/02	+	+	−	−	+	−	−	−	−	−	−	+	−	−	−	−	−	−	−
LOCK 0009	+	+	−	−	+	−	−	−	−	−	−	+	−	−	−	−	−	−	−
LOCK 0004	+	+	+	−	+	+	+	+	+	+	+	+	+	−	+	+	+	+	−
LOCK 0007	+	+	−	−	−	−	−	−	−	−	+	−	−	−	+	+	−	+	−

^1^ Substrates: 1—D-glucose, 2—glycerol, 3—calcium 2-keto-gluconate, 4—L-arabinose, 5—D-xylose, 6—adonitol, 7—xylitol, 8—D-galactose, 9—inositol, 10—D-sorbitol, 11—methyl-αD-glucopyranoside, 12—N-acetyl-glucosamine, 13—D-cellobiose, 14—D-lactose, 15—D-maltose, 16—D-saccharose, 17—D-trehalose, 18—D-melezitose, 19—D-raffinose.
